# Editorial: Neurobiology of sleeping behaviors

**DOI:** 10.3389/fnbeh.2023.1131920

**Published:** 2023-01-17

**Authors:** Emi Hasegawa, Yo Oishi, Daniel Kroeger, Tomomi Tsunematsu, Yves Dauvilliers

**Affiliations:** ^1^Department of Systems Biology, Division of Bioinformatics and Chemical Genomics, Graduate School of Pharmaceutical Sciences, Kyoto University, Kyoto, Japan; ^2^International Institute for Integrative Sleep Medicine (WPI-IIIS), University of Tsukuba, Tsukuba, Japan; ^3^Faculty of Medicine, University of Tsukuba, Tsukuba, Japan; ^4^Department of Anatomy, Physiology, and Pharmacology, College of Veterinary Medicine, Auburn University, Auburn, AL, United States; ^5^Department of Integrative Life Sciences, Graduate School of Life Sciences, Tohoku University, Sendai, Japan; ^6^Creative Interdisciplinary Research Division, Frontier Research Institute for Interdisciplinary Sciences, Tohoku University, Sendai, Japan; ^7^National Reference Centre for Orphan Diseases Narcolepsy Rare Hypersomnias, Sleep Unit, Department of Neurology, CHU Montpellier, University of Montpellier, Montpellier, France; ^8^Institute for Neurosciences of Montpellier (INM), University of Montpellier, INSERM, Montpellier, France

**Keywords:** wakefulness, sleep-wake cycle, sleep disturbances, REM sleep, NREM sleep

Although the interest in the neurobiology of sleep has increased year by year, the functions and control mechanisms of this behavioral state remain relatively unclear (Sehgal and Mignot, 2011, Mander et al., 2017). The fact that virtually all living organisms on earth have sleep highlights the importance of this biological process. Lack of sleep increases the risk of interfering with daily life and developing a variety of diseases. Therefore, it is clear that the physiological function of sleep is very important in maintaining life activities.

Through evolution, the brain and body structure have become more complex, resulting in more diversified patterns and expressions of sleep. The duration of daytime and nighttime sleep, but also its organization over the 24-h varies among animal species. Sleep is usually assessed by electroencephalogram (EEG), which provides an indirect measure of the dense neural network driving sleep and wake states in the brain. Therefore, sleep is altered when normal neurodevelopment is disrupted by internal and external factors. This also provides an opportunity to understand the mechanisms and possible functions of sleep by experimentally modulating internal (e.g., genetics) and external (e.g., drugs) factors. In one recent study, Wang et al. (2022) showed that ubiquitin, which is involved in various biological phenomena such as protein degradation, DNA repair, translation regulation, and signal transduction, is important for neurodevelopment. Shi et al. focused on the ubiquitin ligase gene, Ube3a, and examined its effects on sleep patterns and circadian rhythms. Since the authors found that Ube3a is also expressed in neurons of the suprachiasmatic nucleus (SCN), the center of circadian rhythm, they investigated its effects on circadian rhythm and sleep. They found that under the environment-dependent influence of steady-state light (LL), the mice showed lower activity under in LL. Under LD (light-dark, 12:12 cycles), hourly assessments of wake, non-rapid eye movement (NREM) sleep, and rapid eye movement (REM) sleep revealed that these mice had increased wakefulness, reduced NREM sleep, and REM sleep compared WT mice.

Fujiyama et al. aimed to address the question of “neuronal functions and circuits of sleep” by investigating the contribution of the cerebellum to sleep/wake control. They examined sleep phenotypes in a mouse model in which the midbrain and rhombencephalon region 1 (Ptf1a) genes were conditionally knocked out and the entire cerebellum was deleted. They demonstrate that mice lacking the cerebellum retain relatively intact overall sleep/wake parameters, but that these mice have decreased delta power during NREM sleep and REM sleep time during the dark phase. While the authors acknowledge that this knockout model might also affect a limited number of neurons elsewhere in the brain, the study quite conclusively demonstrates that the overall role of the cerebellum to sleep/wake control is rather limited and thus confirms the number of prior lesion studies.

Geng et al. focused on the relationship between the thyroid system and Restless Leg Syndrome (RLS), a sleep disorder associated with dopaminergic dysfunction. While dopamine controls thyroid systems by downregulation of thyroid stimulating hormone (TSH) and thyroid hormones, the link between thyroid systems and RLS is unclear. They compared thyroid function between RLS patients and healthy subjects and found that RLS patients had significantly higher serum TSH levels, but not T4 or T3 hormone levels, and higher prevalence of subclinical hypothyroidism. They also found that serum TSH levels are higher in good-sleeper patients compared with poor-sleeper patients. Their data indicate the imbalanced thyroid system and sleep patterns in RLS patients, and they hypothesize that the thyroid system may contribute to the development of RLS.

It is not always clear whether altered sleep is the result of an underlying disorder or the cause of the disorder. For example, sleep disturbances are very prevalent in patients suffering from neurodegenerative diseases, but whether sleep is a contributing factor in these diseases is unknown. Minakawa proposed an interrelationship between sleep disorders and neurodegenerative Parkinson's disease (PD) and Alzheimer's disease (AD). Sleep disturbances impact the susceptibility to developing or the progression of PD and AD is resolved by epidemiological and experimental studies. These suggested that the exacerbation of PD and AD pathology is related to negative impact of sleep disturbances as previously reported (Dauvilliers, 2007). However, detecting subtle changes in sleep early in the disease process can sometimes be challenging. Gong et al. investigated the relationship between drug treatment and changes in prefrontal cortex (PFC) functional patterns in patients with chronic insomnia to find new means of detecting sleep disorders in human. They found that functional near-infrared spectroscopy could be a useful tool for assessing sleep status potentially guiding pharmacotherapy. These clinical findings and methods will help clarify the relationship between sleep disorders and neurodegenerative diseases.

As described above, the molecular and neural regulation of sleep-wake and sleep function is gradually becoming clearer, but the physiological significance of sleep is still poorly understood. In addition, the relationship between sleep disorders and neurodegenerative diseases remains to be elucidated. To shed further light on these questions many diverse research reports are needed in addition to those published in this Research Topic. We sincerely hope that this Research Topic will act as a catalyst for future the sleep research.

## Author contributions

All authors listed have made a substantial, direct, and intellectual contribution to the work and approved it for publication.
